# Rhino-Orbital Cerebral Mucormycosis in a Patient With Diabetic Ketoacidosis: A Case Report and Literature Review

**DOI:** 10.3389/fneur.2022.815902

**Published:** 2022-05-04

**Authors:** Nan Dong, Ashly E. Jordan, Xiaozhu Shen, Xuan Wu, Xianghong Guo, Hongru Zhao, Yajuan Wang, Dapeng Wang, Qi Fang

**Affiliations:** ^1^Department of Neurology, The First Affiliated Hospital of Soochow University, Suzhou, China; ^2^Department of Neurology, Suzhou Industrial Park Xinghai Hospital, Suzhou, China; ^3^Independent Research Epidemiologist, New York, NY, United States; ^4^Genoxor Medical Science and Technology Inc., Shanghai, China

**Keywords:** rhino-orbital cerebral mucormycosis, diabetes, metagenomics cell-free DNA next-generation sequencing, orbital apex syndrome, ketoacidosis

## Abstract

**Background:**

Rhino-orbital cerebral mucormycosis (ROCM) is a rare, invasive, and fatal fungal disease. Due to the lack of specific clinical manifestations and adequate auxiliary examinations, patients are easily misdiagnosed in the early stage. Early diagnosis and timely therapy are essential for successful treatment.

**Case Report:**

We report a 68-year-old man with diabetic ketoacidosis, presented with orbital apex syndrome (OAS), fever, and pansinusitis, which progressively worsened to death only 4 days after admission. It was finally confirmed as a fungal *Rhizopus arrhizus* infection by metagenomics cell-free DNA next-generation sequencing (mNGS) testing.

**Conclusion:**

Orbital apex syndrome could be the initial presentation for mucormycosis. Thus, it is necessary to evaluate the presence of mucormycosis in patients with OAS, especially in diabetic or immunosuppressed hosts, and mNGS testing and timely antifungal therapy should be strongly recommended in highly suspected cases.

## Introduction

Mucormycosis is a lethal, angioinvasive fungal disease, which primarily occurs in individuals with an immunocompromised state including uncontrolled diabetes, ketoacidosis, trauma, iron overload, hematological malignancies, and allogeneic stem cell transplantation (1–4). The prevalence of mucormycosis among patients without predisposing medical conditions has also been reported (5). Rhino-orbital cerebral mucormycosis (ROCM) is the most common form of mucormycosis. The incidence of ROCM has been rising worldwide, particularly in India and the Middle East (4, 6, 7). It has recently become a matter of immediate concern in the setting of COVID-19 in India (6). The order Mucorales comprises 261 species in 55 genera, of which 38 are associated with human infection (4). As reported in a global review, the most common responsible agents of mucormycosis are *Rhizopus* spp., *Lichtheimia* spp., and *Mucor* spp., with the most frequent being *Rhizopus* spp. (8). Furthermore, its distribution varies depending on the geographical zones. *Lichtheimia* spp. are the second most frequently isolated agents in Europe and Africa, while in India, the second most commonly isolated agents are *Apophysomyces* spp. (4, 9).

Rhino-orbital cerebral mucormycosis is a rapidly progressive disease, and even a slight delayed diagnosis may lead to devastating consequences. It has been reported that even if intensive antifungal and surgical interventions were achieved, ROCM-related mortality is persistently very high, ranging from 50 to 100% (10). Owing to the lack of specific clinical presentations, the misdiagnosis rate is very high in the early stage. Santosh G Honavar exhibited some warning symptoms and signs in the setting of COVID-19, including nasal stuffiness, foul smell, eyelid, periocular or facial edema, regional pain-orbit, paranasal sinus or dental pain, worsening headache, proptosis, sudden loss of vision, sudden ptosis, facial palsy, and fever (11). We believe that the “red flags” of ROCM mentioned above also apply to other ROCM patients. Thus, any of the over-mentioned symptoms appearing in an immunocompromised patient requires a very high index of suspicion for ROCM. However, in the real world, it has been reported that up to 90% of cases are undiagnosed and untreated (12, 13).

In this case study, we report a 68-year-old man with diabetic ketoacidosis, who had a five-day history of right ptosis, swelling, and pain, and a four-day history of fever, which progressed to consciousness disorder and worsened to death only 4 days after presentation. Metagenomics cell-free DNA next-generation sequencing (mNGS) has confirmed the diagnosis of ROCM in blood and cerebrospinal fluid. Here, we review the literature on the epidemiology, clinical presentation, and advancement in diagnostic and therapeutic approaches of ROCM.

## Case Presentation

In November 2020, a 68-year-old man presented at the emergency department in the First Affiliated Hospital of Soochow University with a five-day history of right ptosis, swelling, and pain, accompanied by a four-day history of fever. He was treated with ceftriaxone, dexamethasone, and low molecular weight heparin (LMWH) at the local hospital for 2 days. However, his condition progressively worsened. He denied headache or convulsion, and his medical history was poorly controlled diabetes.

On neurological examination, the patient presented a complete ophthalmoplegia in the right eye, ptosis, swelling, a fixed dilated pupil unresponsive to light, no light perception, and loss of sensation in the right forehead and eyelids. No purulent nasal discharge, fetor, or anosmia was observed. Meningeal signs were negative, and motor exam revealed normal muscle bulk and tone, power 5/5, and more than two reflexes with normal plantar responses. The sensory exam found that the patient was intact to pinprick and vibration on trunk and limbs. There were no positive signs in the cerebellum.

**Figure 1:**
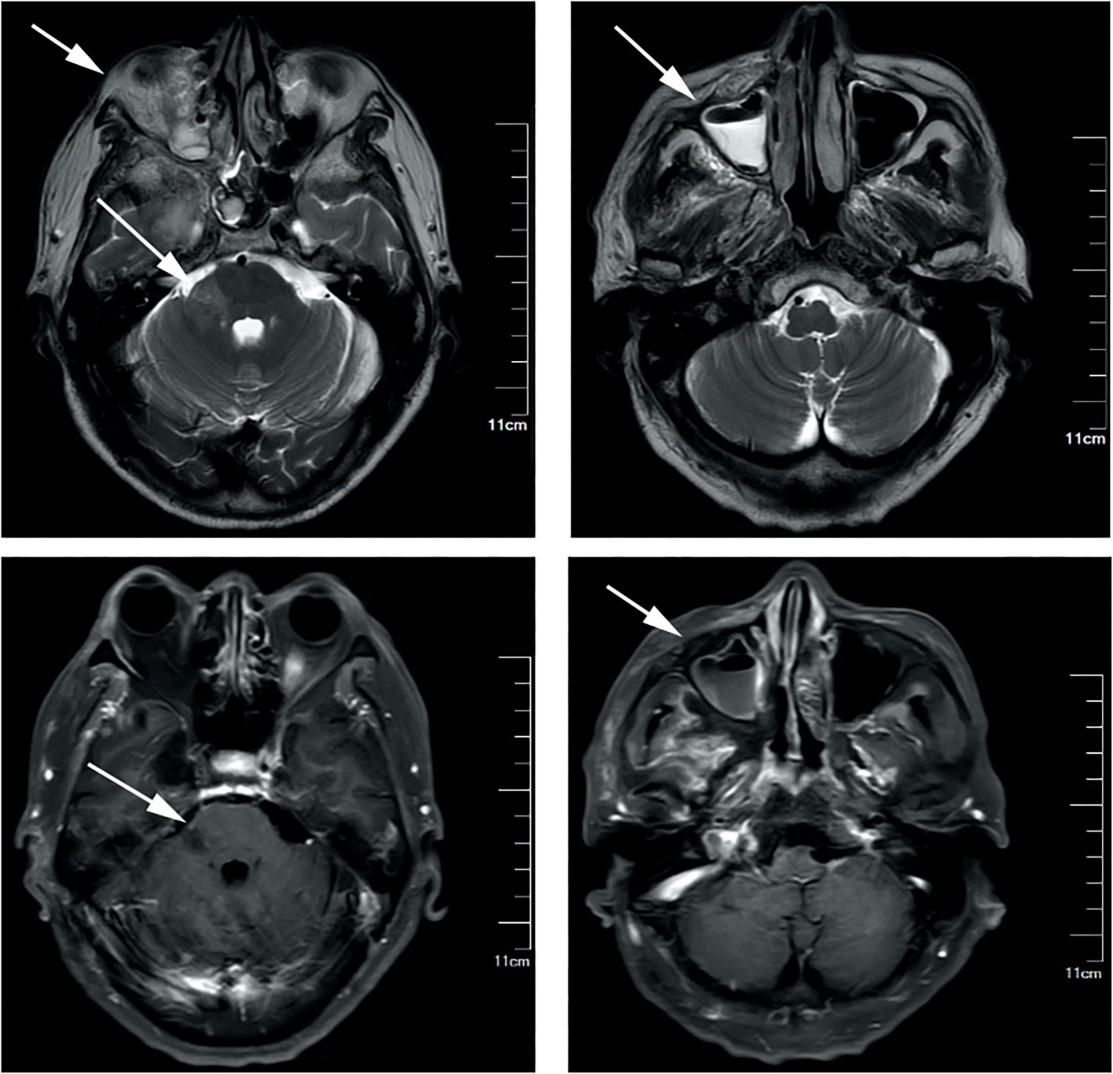
Gadolinium-enhanced brain magnetic resonance imaging (MRI) revealed bilateral pansinusitis and multiple lesions scattered in the right cerebral hemisphere and right pons, showing hypointensity on T1-weighted images, and hyperintensity on T2-weighted and fluid attenuated inversion recovery (FLAIR) images, without contrast enhancement.

On laboratory testing, the capillary glucose was 230 mg per deciliter, and the hemoglobin A1c was 14.6%. Basic hematology showed a white blood cell count of 22,410 per deciliter, 80% of neutrophils, and urinalysis was notable for glucosuria and ketonuria. Cerebrospinal fluid examination revealed elevated white cells of 463 per deciliter (48.2% monocyte), a protein level of 850 mg per deciliter, and glucose of 122 mg per deciliter. Magnetic resonance imaging of the brain revealed pansinusitis and multiple lesions scattered in the right cerebral hemisphere and right pons (Figure 1).

**Figure 2:**
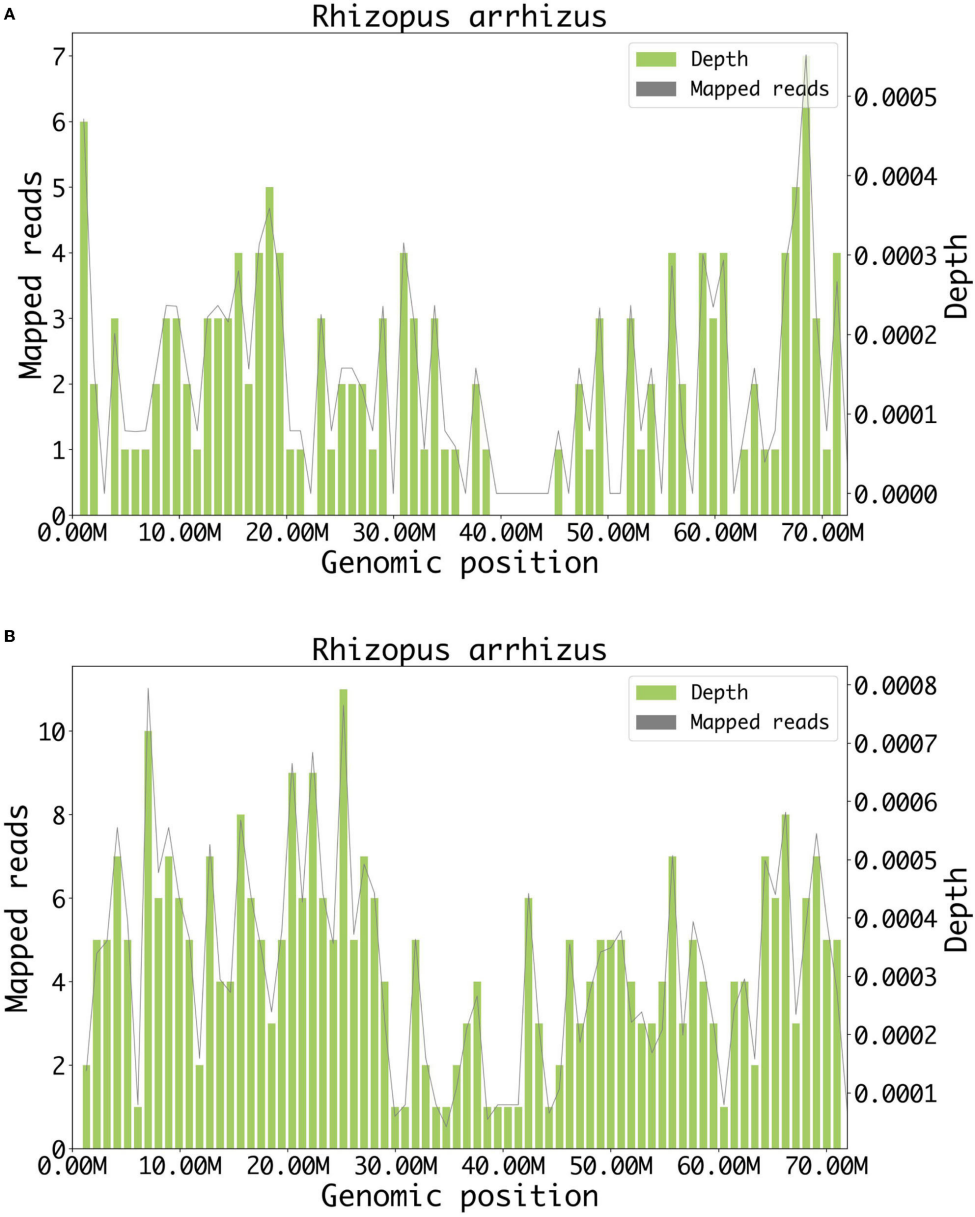
Result of metagenomic NGS. **(A)** A total of 78 DNA reads mapped to *Rhizopus arrhizus* in cerebrospinal fluid. **(B)** A total of 131 DNA reads mapped to *Rhizopus arrhizus* in blood.

The patient presented complete ophthalmoplegia (combination of unilateral impairment of cranial nerves III, IV, and VI), loss of vision (II), and loss of sensation in the right forehead (ophthalmic branch of V). Based on the positive signs above and the imaging results, we located the lesions on the right orbital apex and right brainstem. The etiologies considered included infectious, vascular, inflammatory, neoplastic, and traumatic. In the absence of a definitive diagnosis, the patient was administered ceftriaxone, linezolid, metronidazole, acyclovir, dexamethasone, and LMWH after admission. Due to the rapid development of the patient's condition, the samples of blood and cerebrospinal fluid were immediately sent to Genoxor Medical Science and Technology Inc. for mNGS testing. On the following day, the report indicated that *Rhizopus arrhizus*, which is the most prevalent causative agent of mucormycosis, was detected in both the patient's cerebrospinal fluid and peripheral blood. The reads were 78 in cerebrospinal fluid and 131 in the blood (the sequencing coverage and sequencing depth in Figure 2). The result was verified by quantitative polymerase chain reaction (qPCR) later with the same cerebrospinal fluid sample for mNGS. The qPCR was performed to amplify the specific part of *Rhizopus arrhizus*, with *Rhizopus arrhizus*-specific primers *Rhizopus arrhizus*-F (TTCAAAGAGTCAGGTTGTTTGG) and *Rhizopus arrhizus*-R (CAGTCTGGCTCCAAACGGTTC). Combined with the patient's clinical manifestations, it was finally confirmed to be a fungal *Rhizopus arrhizus* infection. Liposomal amphotericin was timely treated. However, the patient began to experience disturbance of consciousness and unfortunately died within 4 days after presentation.

## Discussion

Champion was the first to propose that a fungal infection involving the cerebellar and orbital sinuses be defined as ROCM, early in 1969 (14). ROCM is a rare yet lethal fungal infection, which is wreaking havoc at an alarming rate in India and other countries (15). In the past few years, the incidence rate of this rare disease has been rising with advances in experimental and imaging systems. However, as ROCM continues to be associated with high mortality, early diagnosis and aggressive treatment are essential for survival. The infrequent presentation of ROCM poses both diagnostic and therapeutic challenges for doctors who are not familiar with the disease. Here, we have presented a case with a fatal ending and discussed the challenging obstacles that lead to high mortality of ROCM from the perspectives of advances in diagnosis and treatment difficulties.

### Epidemiology

It is difficult to measure the incidence of ROCM precisely, since the majority of studies regarding its prevalence originate from case reports, case series, or non-population-based cohort studies rather than population-level studies. Furthermore, the added difficulty in collecting samples and the low sensitivity of etiology tests may underestimate its actual prevalence. In 2017, the authoritative Leading International Fungal Education (LIFE) reprorted that the prevalence of mucormycosis was approximately 910,000 cases annually, almost 98.9% of those were found in India, and the estimated mean mortality was about 38.2% per year (16, 17). Mucormycosis may infect any organ at any age and is classified based on its clinical presentation into the following six clinical forms: rhinocerebral, pulmonary, cutaneous, gastrointestinal, disseminated, and other rare forms (18). A multicenter prospective study, which included 465 subjects with mucormycosis in India, presented that ROCM was the most common (67.7%) presentation, followed by pulmonary (13.3%), cutaneous (10.5%), and other types. The analysis of predisposing factors indicated that diabetes (73.5%) was the dominant factor in all forms of mucormycosis (8). A meta-analysis by W. Jeong identified that ROCM is the most common form in patients with diabetes. In contrast, pulmonary and disseminated forms were more common in those with malignancies and transplantation recipients (19).

### Non-Specific Features of Clinical Presentation

The clinical manifestations of ROCM are related to the location of fungal invasion, which provides clues to lesion location. Among them, bone destruction of the paranasal sinus in the early stage, OAS in the progressive phase, and venous sinus syndrome in the advanced stage are highly suggestive of a potential fungal infection. ROCM is presumed to begin with paranasal sinuses, presenting initially as nasal congestion, headache, fever, or sinus pain, which is indistinguishable from those of more common causes of sinusitis. All sinuses may be involved, and the infection may progressively spread to adjacent structures such as the palate, orbit, and brain tissues within only a few days. Also, there were cases that followed a relatively indolent course progressing over a few weeks (20).

Because of the lack of specific features in the early stage of ROCM, patients often visit ophthalmology, otorhinolaryngology, respiratory, neurology, or other departments. Progressive manifestations are mainly retrobulbar soft tissue inflammation and invade multiple cranial tissues including vessels, optic nerve, the oculomotor nerve, and trochlear nerve, resulting in periorbital pain, eye distension, conjunctival congestion, decreased vision, exophthalmos, and painful ophthalmoplegia like OAS. The appearance of OAS is highly suggestive of a fungal infection. Ursula et al. suggested that when OAS accompanying any sinusitis is observed in a diabetic or immunocompromised patient, a fungal etiology should be suspected (21). Intracranial manifestations often signify a late-stage infection, and intracranial lesions often present on the side of sinusitis. In the pathway of intra-vascular invasion, fungi firstly invade the nasal sinus and intra-orbital vein with abundant blood supply, and further spread by entering the internal carotid artery, surrounded by the venous sinus, forming a fungal embolus, which may embolize in the ipsilateral middle cerebral artery after falling off, resulting in hemiplegia, sensory disturbance, epilepsy, and disturbance of consciousness (22). At the same time, fungi in the venous sinus often invade its surrounding cranial nerves III, IV, V, and VI, presenting ophthalmoplegia and facial pain. In the pathway of a direct invasion through bone lamella, the fungi invade by breaking through the paranasal sinus and orbital apical bone lamella into the brain, resulting in a neurological deficit in the corresponding damaged areas. As the two pathways do not exist independently, the symptoms are complex and variable. However, the trans-vascular approach is relatively common, so venous sinus syndrome indicates that fungal infection has invaded the brain (23).

Due to the close anatomical proximity, it is necessary to distinguish OAS from superior orbital fissure syndrome (SOFS) and cavernous sinus syndrome (CSS). OAS is characterized by the involvement of cranial nerves II, III, IV, VI, and V1 (24). The involvement of the optic nerve differentiates OAS from the other two. In addition, the involvement of sympathetic fibers and the maxillary branch of the trigeminal nerve differentiates CSS from the others (25).

### Advancement in Diagnostic Tests

At present, there are no available circulating clinical biomarkers of ROCM. Definitive diagnosis still depends on conventional diagnostic biopsy, which is invasive and lacks sensitivity and species-level identification (26, 27). Prompt diagnosis of ROCM infection with aggressive antifungal therapy is crucial to increase survival and reduce mortality (28).

In the present case, we used a novel technology to get a rapid and accurate etiological result within 24 h. mNGS has emerged as an effective, non-invasive, and quick laboratory technology. Compared with traditional diagnostic methods, such as histopathological and culture diagnosis, mNGS testing showed better sensitivity to pathogen detection, for its ability to identify a wider range of organism and microbial profiles including uncommon agents, and is less affected by prior antibiotic exposure (29). Armstrong et al. detected 40 plasma samples from at-risk patients for fungal infection, who had pediatric hematology, oncology, or stem cell transplantation. mNGS was used to detect fungal pathogens and was compared to conventional clinical testing. It turned out that mNGS identified fungal pathogens in 7 of 40 patients, among which 66.7% were further proved by biopsy (30).

Metagenomics cell-free DNA next-generation sequencing is a novel promising methodology for invasive fungal infections, supporting rapid and specific detection in various sites of infection. The results of mNGS can become available within 24 h, thereby providing a quick reference for timely diagnosis and antifungal therapy for clinicians (31, 32). Furthermore, in the age of “omics”, mNGS-based approaches can provide more insights into our understanding of various aspects of mucormycosis, including genome structure, biology, determinants of virulence, growth, and metabolism, pathogenicity, drug resistance, and fungus-host interactions, all of which may promote significant progress in the clinical and scientific research of ROCM (33–35). Previous studies indicated that mNGS has a similar sensitivity to specific PCR assays, which is higher than conventional methods (36). However, reduced specificity due to background microbial contamination and inability to distinguish valuable infection or colonization remains challenging for clinicians, emphasizing the significance of cautious interpretation in the context of clinical practice (29).

### Advancement in Therapy

Successful treatment of ROCM requires timely diagnosis, prompt antifungal therapy, and correction of the underlying predisposing factors. Yet, high mortality rates from ROCM infection persist even if all of the above are achieved. There are few prospective randomized controlled clinical trials to evaluate the efficacy of antifungal treatment of ROCM. Global guidelines for diagnosing and managing of mucormycosis in 2019 suggested that intravenous administration of amphotericin B should be considered as the first choice for initial treatment (37). It also stresses that in immunocompromised patients with suspected infection, immediate treatment initiation is strongly recommended. Among the commonly used antifungal drugs, liposomal amphotericin B (L-AMB) is recommended as the first-line antifungal monotherapy for its better brain penetration and less nephrotoxicity (38). Azoles like isavuconazole and posaconazole oral suspensions are also used in first-line treatment, while terbinafine and echinocandins are not effective against mucormycosis (4). Ibrahim et al. demonstrated the superiority of L-AMB/isavuconazonium sulfate combination over either drug alone in treating murine mucormycosis, however, whether this finding could be transformed into humans warrants further investigation (39).

A recent research demonstrates that delayed antifungal therapy significantly increases mortality in patients with mucormycosis when compared with early treatment (40). Except for systemic intravenous medication, topical medication has also been considered valuable. The 2019 guidelines strongly recommend a combination with early complete surgical treatment whenever possible, which should be repeated if necessary (37). Doub et al. reported a unique case of *Rhizopus arrhizus* brain abscess treated with intracavitary amphotericin in the presence of a blood-brain barrier breach of amphotericin B. Unfortunately, the efficacy of this therapy was not ultimately able to be evaluated (41). Safi et al. reported a case of ROCM with focal anterior cerebritis, treated favorably with a retrobulbar injection of deoxycholate amphotericin B and systemic antifungal therapy (42).

Surgical debridement is essential for the successful treatment of ROCM. A study from 13 European countries involving 230 cases of mucormycosis (27% of ROCM) indicated that combination therapy with amphotericin B and debridement achieved a lower mortality rate (24%) than either medication (58%) or surgery alone (44%) (12). Moreover, orbital exenteration is occasionally necessary. Hargrove et al. reported that exenterated patients with fever have a higher survival rate than nonexenterated patients with fever (*p* = 0.0468) (43). Kshitij et al. devised a scoring system to predict the stage when the exenteration is needed, then the scoring system, if validated with 15 cases and turned out to be efficient (44).

Hyperbaric oxygen (HBO) has a fungistatic effect *in vitro* (45), suggesting that it may serve as an adjunctive therapeutic modality for ROCM. Early in 1988, Couch et al. treated two patients with ROCM with adjunctive HBO therapy based on amphotericin B and surgical debridement. A literature review that included 21 patients with ROCM receiving HBO treatment found a high survival rate of 86%, and even higher (94%) when examining outcomes among the diabetic patients (46).

Immunomodulatory therapy including granulocyte colony-stimulating factor (G-CSF), granulocyte-macrophage colony-stimulating factor (GM-CSF), and macrophage colony-stimulating factor (M-CSF) are other medical options to enhance the immune system and aid antifungal activities. Although studies have shown that immunotherapy can improve the prognosis of invasive fungal infection, large-scale randomized controlled trials are needed (47, 48). Recently, Ibrahim et al. discovered a monoclonal anti-ricin B chain antibody named ‘mucoricin,' which may be a promising therapeutic target for mucormycosis (49).

In conclusion, ROCM is a rare, lethal, infectious disease that requires early diagnosis and timely treatment for successful therapy. Clinicians should maintain heightened suspicion for cases with OAS in diabetic or immunocompromised conditions, and pre-emptive antifungal treatment should be recommended in such highly suspicious patients.

## Data Availability Statement

The original contributions presented in the study are included in the article/supplementary material, further inquiries can be directed to the corresponding authors.

## Ethics Statement

The studies involving human participants were reviewed and approved by the Ethics Committee of the First Affiliated Hospital of Soochow University. The patients/participants provided their written informed consent to participate in this study.

## Author Contributions

ND drafted the initial manuscript. ND and AJ contributed equally to this article. All authors were involved in the care of the patient, revised the manuscript and approved the final version.

## Funding

The study was supported by the National Natural Science Foundation of China (82071300), Suzhou Science and Technology Development Plan (SYSD2020073), The Stroke Team of Professor Fang Qi from the First Affiliated Hospital of Soochow University (SZYQTD202106), A follow-up study of cognitive impairment combined with depression in stroke patients (SYSD2020073), and Suzhou Industrial Park Jinji Lake Health Talents (202110).

## Conflict of Interest

YW was employed by Genoxor Medical Science and Technology Inc. The remaining authors declare that the research was conducted in the absence of any commercial or financial relationships that could be construed as a potential conflict of interest.

## Publisher's Note

All claims expressed in this article are solely those of the authors and do not necessarily represent those of their affiliated organizations, or those of the publisher, the editors and the reviewers. Any product that may be evaluated in this article, or claim that may be made by its manufacturer, is not guaranteed or endorsed by the publisher.

## Abbreviations

ROCM: rhino-orbital cerebral mucormycosis
OAS: orbital apex syndrome
mNGS: metagenomics cell-free DNA next-generation sequencing
HBO: hyperbaric oxygen
FLAIR: fluid-attenuated inversion recovery.
